# Myocardial Infarction in Young Adults: A Case Series and Comprehensive Review of Molecular and Clinical Mechanisms

**DOI:** 10.3390/biom15081065

**Published:** 2025-07-23

**Authors:** Bogdan-Sorin Tudurachi, Larisa Anghel, Andreea Tudurachi, Răzvan-Liviu Zanfirescu, Silviu-Gabriel Bîrgoan, Radu Andy Sascău, Cristian Stătescu

**Affiliations:** 1 Internal Medicine Department, “Grigore T. Popa” University of Medicine and Pharmacy, 700503 Iași, Romania; bogdan-sorin.tudurachi@d.umfiasi.ro (B.-S.T.); birgoan_gabi@yahoo.com (S.-G.B.); radu.sascau@umfiasi.ro (R.A.S.); cristian.statescu@umfiasi.ro (C.S.); 2 Cardiology Department, Cardiovascular Diseases Institute “Prof. Dr. George I. M. Georgescu”, 700503 Iași, Romania; leonte.andreea@d.umfiasi.ro (A.T.); zanfirescu_razvan-liviu@d.umfiasi.ro (R.-L.Z.); 3 Pathophysiology Department, “Grigore T. Popa” University of Medicine and Pharmacy, 700503 Iași, Romania

**Keywords:** myocardial infarction, young adults, premature atherosclerosis, nonatherosclerotic mechanisms, cardiovascular risk factors, drugs, spontaneous coronary artery dissection

## Abstract

Acute myocardial infarction (AMI) in young adults, though less common than in older populations, is an emerging clinical concern with increasing incidence and diverse etiologies. Unlike classic atherosclerotic presentations, a significant proportion of AMI cases in individuals under 45 years are due to nonatherothrombotic mechanisms such as coronary vasospasm, spontaneous coronary artery dissection (SCAD), vasculitis, hypercoagulable states, and drug-induced coronary injury. This manuscript aims to explore the multifactorial nature of AMI in young adults through a focused review of current evidence and a series of illustrative clinical cases. We present and analyze four distinct cases of young patients with AMI, each demonstrating different pathophysiological mechanisms and risk profiles—including premature atherosclerosis, substance use, human immunodeficiency virus (HIV)-related coronary disease, and SCAD. Despite the heterogeneity of underlying causes, early diagnosis, individualized management, and aggressive secondary prevention were key to favorable outcomes. Advanced imaging, lipid profiling, and risk factor modification played a central role in guiding therapy. AMI in young adults requires heightened clinical suspicion and a comprehensive, multidisciplinary approach. Early intervention and recognition of nontraditional risk factors are essential to improving outcomes and preventing recurrent events in this vulnerable population.

## 1. Introduction

AMI remains a major cause of death and disability worldwide. While the risk of AMI increases significantly with age, recent trends have highlighted a concerning rise in its incidence among younger adults, typically defined as those under the age of 45 [1,2,3]. Once considered a rarity in this demographic, AMI in the young has garnered increased attention due to its growing prevalence and the disproportionate burden it places on patients, families, and healthcare systems [4,5,6].

Historically, the lower incidence of AMI in younger populations led to less clinical focus on its diagnosis, prevention, and management. However, recent epidemiological data from both national and international registries have revealed a steady increase in hospital admissions for AMI among individuals under 55 years of age [5,7,8,9,10,11]. For instance, data from the Atherosclerosis Risk in Communities (ARIC) Surveillance Study show that nearly one-third of AMI cases now occur in this age group [12].

Although primary prevention strategies and improved risk factor management have contributed to a decline in the overall incidence of AMI in developed countries, recent data suggest a concerning rise in AMI among younger individuals, with an estimated annual increase of approximately 2%. Large cohort studies report that young adults account for 10–12% of all cases of acute coronary syndrome (ACS). The relative prevalence of ST-elevation myocardial infarction (STEMI) versus non-ST-elevation myocardial infarction (NSTEMI) in this population varies across studies. Some cohorts report a higher incidence of STEMI (approximately 45% vs. 40%), while others suggest that NSTEMI predominates (48% vs. 35%). Notably, young patients presenting with STEMI tend to experience a worse short-term prognosis compared to those with NSTEMI, with increased rates of major adverse cardiac and cerebrovascular events (MACCE). In contrast, long-term mortality appears similar between groups; however, after three years, patients with NSTEMI show a higher risk of MACCE. Among prognostic indicators, left ventricular ejection fraction (LVEF) has emerged as the sole independent predictor of long-term mortality. In-hospital mortality, however, is independently associated with reduced EF, the presence of cardiogenic shock, Killip class ≥ 2, and contrast-induced nephropathy. Despite their younger age, individuals with ACS remain at elevated risk for recurrent cardiovascular events and adverse outcomes during long-term follow-up, emphasizing the need for aggressive secondary prevention and close clinical surveillance in this population. This shift has prompted an urgent need to understand the unique risk profiles, etiologies, and clinical characteristics associated with early-onset myocardial infarction [13,14,15,16,17,18].

The predominant cause of AMI in younger patients remains premature atherosclerosis, often driven by traditional cardiovascular risk factors such as smoking, dyslipidemia, hypertension, obesity, and a family history of coronary artery disease (CAD). However, a notable subset of cases—estimated at up to 10%—is due to nonatherosclerotic mechanisms. These include SCAD, coronary vasospasm, thromboembolic events, autoimmune vasculitis, hypercoagulable states, and drug-induced coronary injury. The presence of these diverse mechanisms makes the diagnostic process particularly challenging and can lead to delays in treatment, with serious implications for outcomes [8,19,20,21].

In addition to lifestyle-related contributors, emerging research has identified nontraditional and genetic risk factors with increasing relevance in the young. These include autoimmune diseases, HIV infection, recreational drug use, and heritable conditions such as familial hypercholesterolemia and antiphospholipid syndrome. Genetic predisposition may follow monogenic patterns—as seen in rare syndromes—or may be polygenic in nature, where multiple gene variants interact with environmental exposures. Tools such as polygenic risk scores have shown promise in identifying individuals at higher risk of early cardiovascular events, though their clinical applicability remains under investigation [22,23,24,25,26].

In this review, we present a series of illustrative clinical cases of AMI in young adults and provide a comprehensive review of both the classical and emerging etiologies involved. Through this integrative approach, we aim to enhance awareness of the multifactorial nature of AMI in the young and support clinicians in early recognition, risk stratification, and individualized management strategies for this increasingly vulnerable population.

## 2. Mechanisms of MI in Young Adults

**Figure 1:**
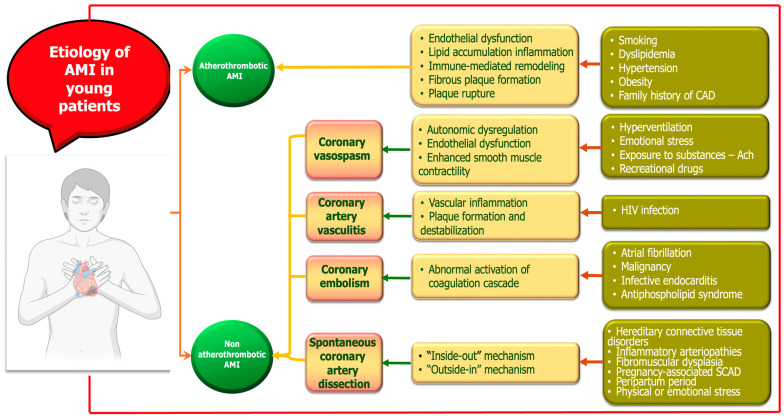
Mechanisms of myocardial infarction in young adults.

AMI in young adults represents a distinct clinical entity with heterogeneous etiologies and pathophysiological mechanisms. While atherosclerotic plaque rupture remains the most common cause, young patients are disproportionately affected by nontraditional and nonatherosclerotic mechanisms (Figure 1). These include drug-induced ischemia, coronary vasospasm, SCAD, coronary embolism, and inflammatory vasculitis. Additionally, hypercoagulable states and autoimmune diseases contribute significantly to myocardial injury in this population. The interplay between genetic predisposition, lifestyle factors, and systemic conditions underscores the complexity of AMI pathogenesis in younger individuals [27,28,29,30,31]. A mechanistic understanding of these diverse etiologies is essential for accurate diagnosis, risk stratification, and tailored therapeutic strategies. In the following sections, we first focus on atheromatous AMI as the principal etiology, followed by a detailed discussion of alternative, nonatherosclerotic mechanisms.

### 2.1. Atherothrombotic MI

Atherosclerosis, a progressive condition that begins in early life, has unique features in younger patients relative to older individuals. It remains the primary cause of AMI in young adults, with plaque rupture responsible for approximately two-thirds of cases [32]. The underlying mechanism involves endothelial dysfunction, lipid accumulation, inflammation, and immune-mediated remodeling, ultimately leading to fibrous plaque formation and potential rupture. Intravascular imaging studies suggest that younger patients generally have less extensive atherosclerosis but greater plaque instability, often presenting with thinner caps and higher rates of rupture or erosion [33,34,35]. Sex-related differences have also been observed, with estrogen potentially exerting a stabilizing effect on plaques in young women [10,11]. Despite these insights, the molecular drivers of premature plaque destabilization remain incompletely understood and warrant further investigation [36,37].

In younger individuals, CAD typically presents with a lower overall burden, including fewer affected vessels, shorter lesion lengths, and smaller plaque volumes. The morphological characteristics of plaques in this group differ significantly, often showing a predominance of fibrous tissue and a lower presence of necrotic or calcified components, along with less evidence of adverse vascular remodeling. High-risk features such as thin-cap fibroatheromas, extensive plaque burden, patchy calcification, and cholesterol crystals are observed less frequently. Despite this, studies have demonstrated a notable incidence of plaque rupture and intraluminal thrombus formation in young patients, indicating a paradoxical tendency toward plaque instability. Sex-based differences are also apparent: young men tend to have more extensive and complex lesions, while young women exhibit thicker fibrous caps and less calcification, supporting the hypothesis of a protective role of estrogen [38,39,40]. Overall, these findings underscore that the pathophysiology of CAD in younger patients is distinct from that seen in older adults, highlighting the importance of age- and sex-specific approaches to diagnosis and management [33,41,42,43].

In young patients presenting with AMI, coronary angiography typically reveals less extensive CAD compared to older populations. Single-vessel involvement is the most frequently observed pattern, with a clear inverse correlation between age and disease complexity. Individuals under the age of 35 most commonly exhibit isolated single-vessel disease, while three-vessel disease is relatively uncommon. The left anterior descending (LAD) artery is the most frequently affected coronary territory, whereas left main (LM) coronary artery involvement is rare. Notable sex differences exist, as young women are more likely to have nonobstructive or single-vessel disease, whereas young men more frequently present with multivessel coronary involvement [2,20,44].

Atherosclerotic plaque characteristics can be assessed using intravascular imaging methods, including intravascular ultrasound (IVUS) and optical coherence tomography (OCT). IVUS employs spectral analysis of radiofrequency backscatter data to generate tissue maps, categorizing plaque into four primary components: necrotic core, dense calcium, fibrous tissue, and fibrofatty tissue. OCT is an imaging modality that employs near-infrared light, offering a thorough visual characterization of plaque through high-resolution imaging [33,45,46]. Vulnerable plaques, precursors to rupture, are typically identified as thin-cap fibroatheromas (TCFAs), featuring a substantial necrotic core enveloped by a thin fibrous cap (less than 65 µm on OCT) [45]. A study of young Indian patients with ACS revealed that fibrous plaques (75%) were the predominant kind, succeeded by macrophage-rich plaques (27%), microchannels (20%), and calcified nodules (14%). Plaque rupture was seen in 31%, plaque erosion in 25%, and lipid-rich plaques in 24%. Patients with premature CAD had a higher prevalence of fibrotic coronary plaques, accompanied by a reduced presence of necrotic and calcified components, in contrast to patients with later CAD [32,33,46].

Risk factors play a central role in the progression of atherosclerosis and MI. Recent studies have identified lipoprotein(a) (Lp(a)) as a significant and independent risk factor for MI in young adults, particularly in those without traditional cardiovascular risk factors. Elevated Lp(a) levels are associated with both atherogenesis and thrombosis, contributing to early coronary events even when LDL cholesterol is within normal limits. Currently, there is no universally accepted threshold for lipoprotein(a) [Lp(a)] levels; however, the European Atherosclerosis Society (EAS) consensus statement suggests that values below 30 mg/dL (or <75 nmol/L) are considered normal, levels between 30 and 50 mg/dL (or 75–125 nmol/L) indicate intermediate cardiovascular risk, and concentrations exceeding 50 mg/dL (or >125 nmol/L) are associated with increased cardiovascular risk and classified as elevated [47]. Moreover, high Lp(a) concentrations have been linked to more extensive CAD, including multivessel involvement, underscoring its role in disease severity and progression. Abnormal apolipoprotein profiles—specifically, increased apolipoprotein B and decreased apolipoprotein A1—further amplify cardiovascular risk by promoting atherogenic particle concentration and plaque vulnerability. These lipid-related markers often go undetected with routine lipid testing, which may limit early diagnosis in younger individuals. Importantly, Lp(a) is largely unaffected by statins or lifestyle changes, necessitating alternative therapeutic strategies. Given these associations, comprehensive lipid profiling, including Lp(a) and apolipoprotein measurements, should be considered in the cardiovascular risk assessment of young patients presenting with ACS [48,49,50,51].

In patients with STEMI undergoing percutaneous coronary intervention (PCI), certain plaque features—such as lower thrombus burden, absence of rupture, and lipid-rich content—have been associated with better post-procedural microvascular perfusion [52,53]. Conversely, plaque rupture has been implicated in an increased risk of no-reflow or slow-flow complications following PCI [54]. Clinical outcomes may vary between individuals with ACS resulting from plaque rupture and those with an intact fibrous cap [32,54].

Although there are no specific clinical guidelines tailored exclusively to the management of AMI in younger patients, current practice generally mirrors the therapeutic strategies used in older populations. Standard treatment typically includes dual antiplatelet therapy (DAPT), beta-blockers, angiotensin-converting enzyme inhibitors (ACEIs) or angiotensin receptor blockers (ARBs), and statins. However, evidence suggests that younger patients are less likely to undergo coronary angiography and PCI compared to slightly older cohorts. Notably, young women are consistently treated with PCI, DAPT, and statins at lower rates than men, highlighting a potential sex-based disparity in the application of evidence-based cardiovascular therapies. These differences have been consistently reported in several observational studies and national registries [12,20,44].

Furthermore, current revascularization guidelines do not differentiate based on patient age. In STEMI, primary PCI remains the preferred intervention, with coronary artery bypass grafting (CABG) typically reserved for patients with extensive myocardial jeopardy or those unsuitable for PCI. For NSTEMI, PCI is commonly employed in cases of single- or two-vessel disease, whereas CABG is favored in patients with complex multivessel disease, particularly involving the LAD or LM coronary artery. In younger individuals, the choice between PCI and CABG requires careful consideration of both short-term recovery benefits and long-term durability, as well as the likelihood of future revascularization procedures [55,56,57,58].

### 2.2. Nonatherothrombotic MI

While atherosclerosis remains the predominant cause of MI, a significant proportion of cases in young individuals are attributed to nonatherothrombotic mechanisms. These include coronary artery anomalies, vasospasm, SCAD, coronary artery vasculitis, and prothrombotic states, which often present diagnostic and therapeutic challenges. Recognizing these alternative etiologies is essential for accurate diagnosis and appropriate management, as they often require different therapeutic approaches than atherosclerotic MI [6,11,59].

#### 2.2.1. Coronary Vasospasm

Coronary vasospasm is an important but often underrecognized cause of MI in young patients, frequently classified within the spectrum of myocardial infarction with nonobstructive coronary arteries (MINOCA). MINOCA is characterized by myocardial infarction indicated by elevated cardiac biomarkers, occurring without significant coronary artery obstructions, with causes that include both atherosclerotic plaque events and nonatherosclerotic factors such as coronary artery dissection. It is defined by transient, intense coronary artery constriction leading to significant ischemia and ECG changes, often in the absence of fixed atherosclerotic lesions. Coronary microvascular dysfunction (CMD) is a nonatherosclerotic cause of MINOCA characterized by structural and functional abnormalities of the coronary microcirculation. It is commonly associated with cardiovascular risk factors and contributes to adverse clinical outcomes. Pathophysiological mechanisms include heightened microvascular vasoconstriction, impaired endothelium-dependent and -independent vasodilation, increased microvascular resistance due to capillary rarefaction, and extramural compression. CMD often presents with angina, exertional dyspnea, or heart failure symptoms, despite normal findings on physical examination and standard angiography. Diagnosis relies on clinical assessment and noninvasive evaluation of coronary flow reserve (CFR) and myocardial blood flow at rest and stress. However, CMD can only be definitively diagnosed after excluding obstructive coronary artery disease. Invasive coronary angiography, combined with intracoronary physiological testing, offers a comprehensive approach by assessing both epicardial and microvascular function [60,61,62,63].

Management is patient-centered and involves optimizing comorbid conditions. Treatment remains challenging due to limited guideline-based therapies and frequent persistence of angina despite standard antianginal medications. Therefore, therapy should be tailored to the underlying pathophysiologic mechanism whenever possible. Vasospasm may occur spontaneously or be provoked by various triggers, including hyperventilation, emotional stress, or exposure to substances such as acetylcholine and ergotamine. Notably, drug-induced vasospasm—particularly from stimulants like cocaine or amphetamines—is a well-established precipitant, especially in younger populations. The underlying mechanisms include autonomic dysregulation, endothelial dysfunction, and enhanced smooth muscle contractility [28,59,64]. Diagnosis may require provocative testing, as angiography can appear normal once the spasm resolves. Management involves the use of calcium channel blockers and nitrates, alongside strict avoidance of smoking and vasospasm-inducing agents. While recurrence is possible, long-term outcomes are generally favorable with appropriate therapy and lifestyle changes [59].

Recreational drug (RD) use is an increasingly prevalent public health concern, particularly in high-income countries, where it contributes to rising healthcare costs and a decline in overall life expectancy. Among young individuals, substance abuse is associated with a higher incidence of premature cardiovascular events, including MI. Numerous mechanisms contribute to RD-associated ACS, including sympathetic nervous system activation, which raises cardiac oxygen demand and decreases cardiac oxygen delivery due to coronary vasospasm [65,66]. Other pathophysiological mechanisms that have been implicated include endothelial dysfunction, accelerated atherosclerosis, plaque rupture, thrombosis, and direct toxic effects on the myocardium; however, these are dependent on the patient’s underlying cardiovascular condition as well as the particular drug in question.

The ADDICTO-USIC study, a prospective multicenter investigation in France, reported that over 10% of patients admitted to coronary intensive care units had recently used recreational drugs. Similarly, the ADDICT-ICCU study found that 12% of patients presenting with MI and no standard modifiable cardiovascular risk factors (SmuRF-less) tested positive for psychoactive substances. Among individuals with STEMI, 6% had positive toxicology screens at admission, with cannabis being the most frequently detected drug. Acute drug use was independently associated with worse in-hospital outcomes, including higher rates of mortality, cardiogenic shock, and resuscitated cardiac arrest. Notably, reliance on self-reported substance use may lead to the underestimation of actual prevalence in this population [67,68,69].

There is growing concern over the rising incidence of MI in young adults, with recreational drug use identified as a significant contributing factor. A retrospective study in Amsterdam found that 24.9% of patients aged 18–50 presenting with ACS reported illicit drug use, predominantly cannabis (16.2%) and cocaine (4.8%). These individuals were primarily young males and had high rates of tobacco use. Drug users demonstrated more extensive myocardial damage and reduced left ventricular (LV) function compared to nonusers. Another study focusing on very young patients (ages 18–24) reported intravenous drug use in 14.9% and cannabis use in 12.8% of those with MI. These findings highlight the need for routine substance use screening and targeted prevention strategies in the management of young patients with acute coronary events [65,70].

Cannabis is one of the most used recreational drugs globally, including in Europe, where it remains an illicit substance in countries like France. Registry data from France show a higher prevalence of cannabis use among young adults hospitalized for AMI, particularly in the 18–24 age group. When inhaled or smoked, its effects appear rapidly and typically last two to four hours, making this route more prevalent among users. The primary active compounds—Δ9-tetrahydrocannabinol and cannabidiol—mediate complex cardiovascular effects that are not yet fully understood. Acute cannabis use has been associated with increased sympathetic activity, endothelial dysfunction, reduced parasympathetic tone, and enhanced platelet aggregation, all of which contribute to elevated myocardial oxygen demand, prothrombotic states, and vascular instability. These mechanisms may explain the significantly elevated risk of AMI observed shortly after cannabis use, with studies reporting up to a fivefold increase in MI risk within the first hour post-consumption. While some studies have not found significant differences in in-hospital outcomes between cannabis users and nonusers, cannabis use has been linked to an increased incidence of arrhythmias, cardiac arrest, and other serious cardiac events. Notably, MI can occur even in young cannabis users without prior angina, hypertension, or atherosclerotic disease, underscoring the need for greater awareness of cannabis-related cardiovascular risks in this population [23,67,68,69,70,71,72,73,74,75,76].

Cocaine significantly increases the risk of AMI, particularly within the first few hours after use—up to 24-fold compared to nonusers. Intense vasoconstriction, resulting from norepinephrine and dopamine reuptake inhibition, may induce coronary artery spasms that diminish or entirely restrict blood supply to the myocardium, thus causing a myocardial infarction. It also enhances heart rate and blood pressure, hence increasing myocardial oxygen demand. Cocaine use is a major contributor to nonfatal MI in young individuals and is commonly associated with emergency presentations for nontraumatic chest pain. Its cardiovascular impact includes accelerated atherosclerosis, aortic dissection, and cardiomyopathy, while cerebrovascular complications such as ischemic and hemorrhagic strokes are also reported. Diagnosis can be challenging, especially as effects may persist for up to 72 h post-use. Management includes nitrates and benzodiazepines for chest pain, while beta-blockers are generally avoided due to the risk of unopposed alpha-adrenergic stimulation [23,65,67,69,70,71,75,77,78,79].

Amphetamines, including methamphetamine and MDMA (ecstasy), are potent psychostimulants that increase synaptic concentrations of dopamine, norepinephrine, and serotonin, leading to tachycardia, hypertension, and vasoconstriction. Their use is associated with serious cardiovascular complications such as MI, heart failure (HF), arrhythmias, and methamphetamine-induced cardiomyopathy. These substances can also provoke cerebrovascular events, including both ischemic and hemorrhagic strokes, as well as aortic dissection—potentially at a higher rate than cocaine—due to recurrent hypertensive surges and vascular injury. MDMA has been reported to trigger serotonin syndrome and exert proarrhythmogenic effects. Chronic use may lead to dilated cardiomyopathy and increased long-term cardiovascular risk. In rare instances, other recreational agents like nitrous oxide have also been linked to ACS, possibly through endothelial dysfunction [65,67,69,75,77,78].

Intravenous opioid use is a major risk factor for infective endocarditis, particularly affecting the right-sided heart valves, and is associated with high morbidity and mortality in young adults. Overdose-related complications include aspiration pneumonia and, in cases of the repeated injection of crushed tablets, pulmonary talcosis due to embolization of insoluble fillers, leading to granulomatous lung disease. Opioid intoxication, especially with methadone, has arrhythmogenic potential through QT interval prolongation and may precipitate torsades de pointes via inhibition of cardiac I_Kr potassium channels. Methadone use carries a recognized risk of serious ventricular arrhythmias. Opioid use disorder has also been linked to adverse outcomes in patients hospitalized with AMI. These findings underscore the need for cardiovascular monitoring and infection prevention strategies in individuals who misuse opioids [23,67,69,71,77,78,80].

#### 2.2.2. Spontaneous Coronary Artery Dissection

SCAD is a nontraumatic, noniatrogenic cause of ACS, often underdiagnosed despite its clinical relevance. It involves the formation of an intramural hematoma within the coronary artery wall, which can compress the true lumen and lead to myocardial ischemia or infarction. Two main pathophysiological mechanisms have been proposed. The “inside-out” mechanism begins with an intimal tear, allowing blood to enter and expand a false lumen within the medial layer. The “outside-in” mechanism involves bleeding from the vasa vasorum into the vessel wall without an intimal rupture. In both scenarios, the expanding hematoma or dissection flap can obstruct coronary blood flow, leading to clinical manifestations of ACS [81,82].

The incidence of SCAD among patients presenting with ACS or undergoing coronary angiography ranges from 0.12% to 4%, depending on the study design and population. SCAD predominantly affects women under the age of 50 and may account for up to 24% of ACS cases in this group. While less common, men can also be affected, often presenting at a younger age with different clinical profiles, as observed in the CanSCAD study. Traditional cardiovascular risk factors are typically uncommon, though hypertension is reported in approximately 45% of SCAD cases. Meta-analyses indicate a MACCE rate of 7.8 per 100 person-years and a recurrence rate of 5.5 per 100 person-years, emphasizing the importance of long-term follow-up in this population [83,84,85,86,87].

The underlying causes of SCAD are not fully understood, but the condition is thought to arise in individuals with a genetic or acquired vulnerability to coronary artery wall abnormalities. Hereditary connective tissue disorders—such as Marfan syndrome, Ehlers–Danlos syndrome, and Loeys–Dietz syndrome—as well as inflammatory arteriopathies have been implicated. Fibromuscular dysplasia (FMD) is the most frequently associated vascular condition, present in up to 72% of SCAD cases. Pregnancy-associated SCAD (P-SCAD) is a major cause of AMI in young women and accounts for up to 43% of pregnancy-related AMI events. Additional risk factors include the peripartum period, multiparity, hormonal therapies, systemic inflammation, and significant physical or emotional stress. Emerging evidence also suggests a potential link between SCAD and COVID-19 infection [88,89,90].

The genetic basis of spontaneous coronary artery dissection is an emerging area of research. There is a well-documented association between SCAD and genetic factors linked to FMD, with familial cases supporting a hereditary predisposition. Recent studies have identified potential genetic contributors, including variants in genes such as TLN1 and TSR1, as well as common susceptibility loci near PHACTR1 and ADAMTSL4. These findings suggest a polygenic influence on SCAD risk. Additionally, certain monogenic disorders—such as inherited dyslipidemias and metabolic syndromes—are associated with early-onset CAD and may overlap with SCAD pathophysiology [88,91,92,93,94,95].

SCAD commonly presents as ACS, including both STEMI and NSTEMI. Early recognition is essential, as SCAD patients typically receive less PCI and demonstrate significantly lower in-hospital mortality compared to ACS patients without SCAD. Coronary angiography remains the first-line diagnostic tool, though it cannot visualize the arterial wall, making it less definitive in ambiguous cases. Intravascular imaging modalities such as OCT or IVUS can provide detailed structural information but are used cautiously due to potential risks, especially with OCT. Coronary computed tomography angiography (CCTA) is increasingly used for follow-up and for identifying associated vascular abnormalities, although normal CCTA does not rule out SCAD. Angiographically, SCAD is categorized into four types based on lesion appearance, with type 2 being the most common [83,84,88,96,97,98,99,100,101].

The management of SCAD is individualized and may involve conservative, medical, or interventional strategies. In hemodynamically stable patients without ongoing ischemia, a conservative approach is generally preferred, allowing for spontaneous vessel healing, as documented in follow-up angiographic studies. However, invasive intervention is warranted in high-risk cases, such as those with ongoing ischemia involving a large myocardial territory, STEMI, cardiogenic shock, life-threatening ventricular arrhythmias, or dissection of the LM coronary artery. These patients require prompt evaluation and tailored therapeutic decisions to optimize outcomes [88,96].

Beta-blockers are commonly used in SCAD for their protective effects, while aspirin and DAPT are often prescribed, though the ideal duration of DAPT remains unclear. Emerging evidence supports a more conservative antiplatelet approach in select patients. The role of anticoagulation is debated and should be individualized, as it may worsen intramural hematoma. Statins are not routinely indicated but may be used in patients with coexisting dyslipidemia or for their pleiotropic benefits [84,91,93,96,102].

PCI is reserved for SCAD cases with hemodynamic instability, ongoing ischemia, or LM coronary artery involvement, with drug-eluting stents commonly utilized. Alternatives like bioresorbable scaffolds and cutting balloon angioplasty have been explored in selected cases. PCI is more frequently performed in younger patients and those with P-SCAD, LM or LAD dissection, and multivessel disease. However, the risk of iatrogenic dissection during intervention is significant, and CABG is rarely needed, typically in complex or failed PCI cases. Revascularization has not been shown to reduce long-term recurrence. Cardiac rehabilitation plays a critical role in recovery, addressing both physical and psychological aspects, as many patients experience persistent chest pain, anxiety, or depression despite generally favorable long-term outcomes [91,93,95,96,102,103].

Despite recent guideline updates, a lack of large-scale prospective studies and randomized trials leaves many aspects of SCAD’s clinical course and outcomes unresolved. While long-term survival is generally favorable, the risk of MACCE remains significant. P-SCAD is associated with higher in-hospital, 30-day, and 3-year event rates, suggesting a more severe clinical profile. Factors such as FMD and genetic disorders have been identified as independent predictors of recurrence. Beta-blocker use may reduce recurrence risk, while hypertension and FMD have been linked to higher recurrence rates in meta-analyses. Observational data also highlight persistent infarct size and 30-day readmission as important indicators of prognosis, emphasizing the need for further research to refine long-term management strategies [83,86,88,89,93,104,105].

#### 2.2.3. Coronary Artery Vasculitis

**Figure 2:**
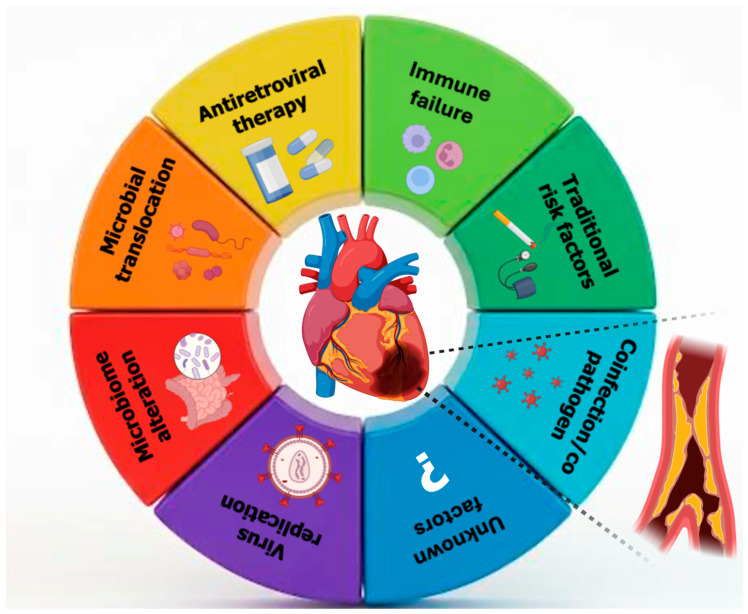
Multifactorial mechanisms linking human immunodeficiency viruses to coronary artery disease in young adults.

Coronary artery vasculitis (CAV) is a rare but clinically significant cause of MI in young individuals, particularly in those without traditional cardiovascular risk factors. The pathogenesis involves immune-mediated inflammation and cytokine-driven damage to the vascular wall, which can lead to luminal narrowing, thrombosis, or aneurysmal changes in the coronary arteries [106]. HIV infection has emerged as a nontraditional risk factor for cardiovascular disease, including MI, particularly in young adults. The increased cardiovascular risk in this population is multifactorial, involving chronic systemic inflammation, immune activation, endothelial dysfunction, and a higher prevalence of traditional risk factors such as smoking and dyslipidemia. Moreover, HIV-associated vasculitis, a form of secondary inflammatory arteriopathy, may contribute to coronary artery involvement and ACS in the absence of significant atherosclerosis. Like other forms of coronary artery vasculitis, HIV-related vascular inflammation may present as unexplained AMI, arrhythmias, or HF in young individuals without evident cardiovascular risk factors (Figure 2). Given the underrecognized nature of HIV-associated coronary involvement, a high index of suspicion is essential for timely diagnosis and management [32].

The worldwide incidence of HIV-related cardiovascular diseases has increased thrice in the past twenty years. It has been found that people living with HIV (PLWH) are more likely to have an MI due to atherosclerosis. Their risk is 2.57 (95% CI, 2.30–2.86) per 1000 person-years, compared to 1.30 (95% CI, 1.09–1.56) per 1000 person-years for people who are not HIV [107,108]. Moreover, those with detectable viremia have greater MI risks than those without, indicating a biological gradient. HIV RNA levels below 500 copies/mL increased MI risk by 1.39-fold, whereas those over 500 copies/mL increased it by 1.75-fold. Lower CD4+ cell counts, indicating immunological progression and partial recovery from HIV, and histories of prolonged viral replication have been linked to increased MI risk in PWH [109]. Individuals with HIV frequently have ACS 10 years sooner than those without HIV. In MI patients, one-month mortality might reach 20%. Furthermore, HIV+ individuals had a six times higher risk of ACS recurrence within 12 months after the original occurrence. Excess ACS recurrence rates are mostly caused by unstable angina episodes caused by new coronary artery obstructive lesions from accelerated atherosclerosis [110]. On the other side, HIV-infected women had double the risk of MI compared to males.

**Figure 3:**
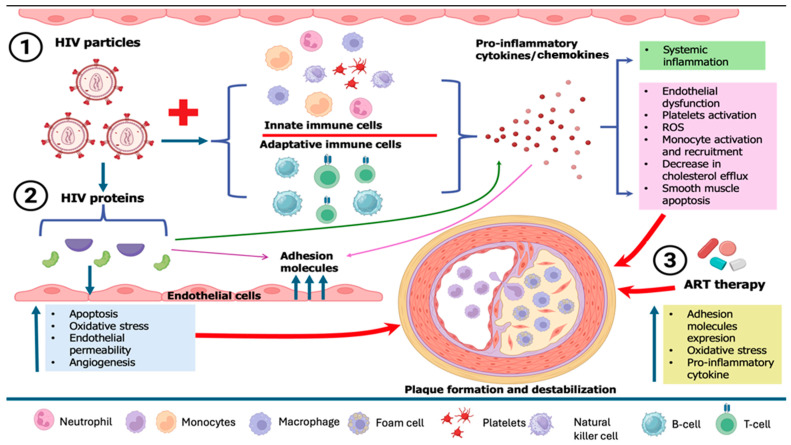
Pathophysiological mechanisms of HIV-related plaque formation and destabilization. ART, antiretroviral therapy; HIV, human immunodeficiency virus.

HIV-related cardiovascular diseases have become a predominant source of morbidity among people living with HIV, driven by endothelial dysfunction, elevated prothrombotic activity, high prevalence of traditional cardiovascular disease (CVD) risk factors, inflammation brought on by HIV infection or residual viremia (which persists despite antiretroviral therapy (ART)), and the adverse effects of ART on the metabolic profile [111,112]. Endothelial dysfunction, which triggers immunological activation, is the first stage of atherogenesis. The recruitment, migration, and transformation of macrophages into foam cells are caused by this inflammation in the endothelium, which is mainly mediated by IL-6 and monocyte chemoattractant protein-1 (MCP-1), two proteins that are often increased in HIV infection [113]. Monocytes, macrophages, and CD8 cells are activated by the PLWH immune response, which results in the release of profibrotic and proinflammatory cytokines that are responsible for chronic inflammation, hypercoagulation, excessive collagen production, vascular thrombosis, vascular endothelial dysfunction, hyperlipidemia, left ventricle (LV) dysfunction, and fibrotic remodeling [114]. Furthermore, in chronic HIV infection, there is a tendency for macrophages to differentiate preferentially into M1-type macrophages, which may elevate levels of cholesterol and destabilize the fibrous cap, in contrast to M2-differentiated macrophages that may enhance plaque instability. Factors that might augment this preferred M1 differentiation in HIV include soluble CD14 and soluble CD163 proteins, both of which are increased in HIV. Additionally, macrophages facilitate platelet aggregation, perhaps resulting in coronary obstruction in ACS. Consequently, the heightened activity of macrophages accelerates the development of arterial plaques, enhances their fragility, and likely exacerbates their harmful consequences upon rupture in individuals with HIV [115] (Figure 3).

Increasing data indicates that individuals with HIV, regardless of their ART status, have subclinical atherosclerosis. People with HIV in the Multicenter AIDS Cohort Study (MACS) had a much higher prevalence of noncalcified arterial plaque compared to matched uninfected controls, even after controlling for conventional atherosclerotic cardiovascular disease (ASCVD) risk factors. Individuals with HIV exhibited 1.4 times higher chances of experiencing >50% stenosis of a coronary artery in this research, and decreased CD4 counts correlated with an increased probability of >50% stenosis, irrespective of demographic variables (albeit not established ASCVD risk factors). Arterial inflammation is more prevalent in HIV-positive individuals compared to matched healthy groups [116]. HIV+ men have higher rates of CAD (59% vs. 34%), larger plaque volumes (55.9 μL vs. 0 μL), more plaque-forming segments (1 vs. 0 segments), and higher Agatston coronary calcium scores (46% vs. 25%), despite a similar Framingham 10-year risk and family history. In addition, HIV infection duration is linked to coronary plaque volume and segment count. The correlations remain substantial after adjusting for age and other risk variables. Moreover, indinavir, lopinavir-ritonavir, abacavir, and didanosine are linked to a higher risk of MI [117,118]. HIV+ women face disparities in healthcare access due to factors such as increased tobacco use, reduced ART effectiveness, immune activation, systemic inflammation, higher nonwhite populations, and lower economic status. HIV+ women had higher levels of inflammatory chemokine CXCL10, CD163 macrophages, and CD14+ CD16+ monocytes after immune activation compared to HIV+ males. Additionally, HIV+ women have a higher risk of noncalcified plaques in their coronary arteries, which might rupture or erode [111,112].

A recent single-center study conducted on young patients with confirmed HIV-1 infection, undergoing ART and exhibiting undetectable viremia, revealed that over a quarter of PLWH with undetectable viremia presented with subclinical atherosclerosis in either the coronary or carotid arteries [108]. Conventional risk factors associated with an increased likelihood of cardiovascular disease include smoking, gender, and age. A meta-analysis revealed that age did not influence the relative risk of cardiovascular disease in people living with HIV, suggesting that younger individuals should not be overlooked. Age specifically increased the relative risk of dyslipidemia. Nevertheless, young individuals are more prone to developing CAD, maybe owing to extended working hours and increased financial stress. Society, stigma, and smoking may increase men’s risk of cardiovascular disease, which can accelerate its progression [119].

Concomitant comorbidities, including hepatitis C virus and microbial translocation, further exacerbate inflammation and the risk of atherosclerotic disease. Certain ART regimens may potentially partially contribute to atherosclerotic disease via dyslipidemia and insulin resistance [120].

Dysregulated lipid profiles are prevalent in people with HIV and are often characterized by reduced high-density lipoprotein cholesterol (HDL-C) levels and increased triglyceride levels in both treated and untreated individuals. HIV impairs HDL function, and antiretroviral therapy does not restore HDL levels to pre-infection baselines. The impact of ART on lipid levels differs by class and individual medication. Some types of protease inhibitors (Pis), especially older versions, raise the levels of total cholesterol, low-density lipoprotein cholesterol (LDL-C), and triglycerides. On the other hand, nucleoside and non-nucleoside reverse transcriptase inhibitors (NRTIs and NNRTIs) have more variable effects [113].

HIV-positive patients hospitalized for MI exhibit reduced adherence to AHA/ACC and ESC guideline-directed care, prolonged hospital stays, increased healthcare costs, and a lower likelihood of receiving anti-platelet, antihypertensive, or lipid-lowering therapies compared to HIV-negative patients [110,121].

Women and men with HIV have a higher incidence of Type 2 myocardial infarction (T2MI) than Type 1 MI (T1MI). T2MI in patients is caused by increased or reduced myocardial oxygen demand, commonly owing to bacteremia, illicit drug use, hypertensive emergencies, and respiratory distress or failure. T2MI patients, mostly black, are less likely to regularly use ART compared to T1MI patients. The rupture or erosion of atherosclerotic plaque causes Type 1 MI in older HIV+ individuals, often white men with comorbidities such as hypertension and renal failure. Compared to T1MI patients, HIV+ individuals with MI had considerably higher death rates following T2MI [122].

A research on the mortality rate of atherosclerotic cardiovascular events in HIV-positive individuals with optimal adherence to ART found that ACS prevalence was greater even with excellent adherence. Mortality rates following ACS were similar to those in the HIV-negative group [123].

In individuals living with HIV, some antiretroviral therapy classes may induce insulin resistance, hyperglycemia, and dyslipidemia. ART may increase carotid intima-media thickness, cause stenosis in carotid and coronary arteries, and hinder vascular flow-mediated dilatation. This underscores the need for judicious ART selection predicated on individual cardiovascular risk, since managing HIV while mitigating cardiovascular risks is complex [110].

PLWH must be carefully managed following revascularization due to hemorrhagic risks and antiretroviral sequelae. New studies recommend statins or antiplatelet drugs for PLWH, highlighting the benefits of stringent lipid-lowering therapy in this group. Post-procedural changes in ART regimens may be needed to prevent cardiovascular risk [124]. Medical stabilization and coronary angiography with stent placement must occur within 24 h after admission for individuals with NSTEMI. Patients with STEMI are advised to have PCI with stent placement and antiplatelet therapy for PLWH within 12 h of symptom onset. Stent implantation and percutaneous coronary intervention are indicated for individuals with persistent symptoms beyond 12 h, as well as for those experiencing cardiac arrhythmias, hemodynamic instability, recurrent chest pain, or HF. In those with complex CAD undergoing ART without advanced immunosuppression, CABG surgery is both safe and effective, exhibiting comparable inpatient mortality rates but slightly increased post-operative blood transfusion requirements relative to HIV-negative patients [122]. However, the risk of AMI seems to be independent of the harmful metabolic effects of antiretroviral medication and conventional atherosclerotic risk factors. Consequently, HIV infection may be regarded as a significant risk factor for MI in young individuals, alongside relatively rare adult syndromes such as rheumatoid arthritis, systemic lupus erythematosus, and obstructive sleep apnea, akin to traditional risk factors like hypertension and smoking [21].

A large HIV cohort study investigating MI types by age revealed that T1MI occurred in adults across all age groups among PLWH. T2MI constituted over fifty percent of all MIs and manifested at a greater frequency than T1MI until the age of 40. This contrasts with studies of the general population, which show that T2MI accounts for a far lower percentage of MIs and usually affects older people [125]. To better illustrate the diverse etiologies, clinical features, and management strategies of AMI in young adults, Table 1 summarizes the key characteristics, highlighting the diagnostic pathways, underlying mechanisms, and therapeutic approaches.

## 3. Case Series

### 3.1. Case 1

**Figure 4:**
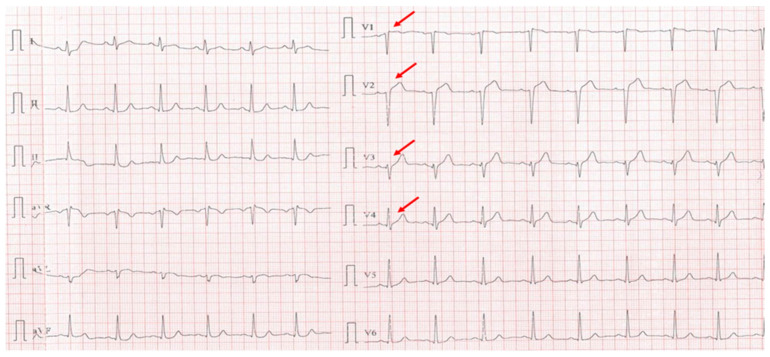
Electrocardiogram with anterior STEMI (red arrows).

A 40-year-old sedentary male, presented with a significant anginal episode four hours after intense physical exertion (Figure 4). He is a nonsmoker but reports high levels of occupational stress. He had a known history of elevated LDL-C and was on atorvastatin 20 mg daily; however, adherence to the therapy was inconsistent. His family history is notable for maternal type 2 diabetes mellitus. On admission, the patient was hemodynamically and respiratory stable, with a blood pressure of 115/65 mmHg, a heart rate of 105 bpm, and oxygen saturation (SpO_2_) of 94%. Laboratory investigations revealed elevated cardiac enzymes and an LDL-C of 188 mg/dL, while everything else was within normal parameters.

**Figure 5:**
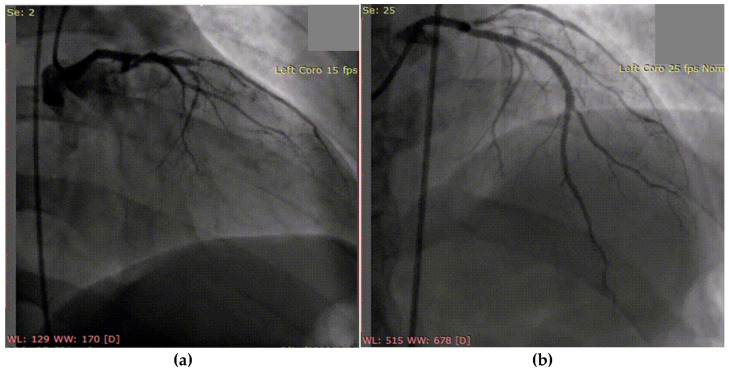
Coronary angiography of the left coronary system: (**a**) acute thrombotic occlusion of the left anterior descending artery; (**b**) subocclusion of the marginal artery.

Following successful coronary angioplasty with drug-eluting stent placement for acute thrombotic occlusion of the left anterior descending artery (Figure 5), the patient was initiated on optimal post-AMI medical therapy. This included dual antiplatelet therapy (aspirin and ticagrelor), high-dose statin and ezetimibe (atorvastatin 80 mg and ezetimibe 10 mg/day), an ACE inhibitor (zofenopril), and a beta-blocker (bisoprolol).

**Figure 6:**
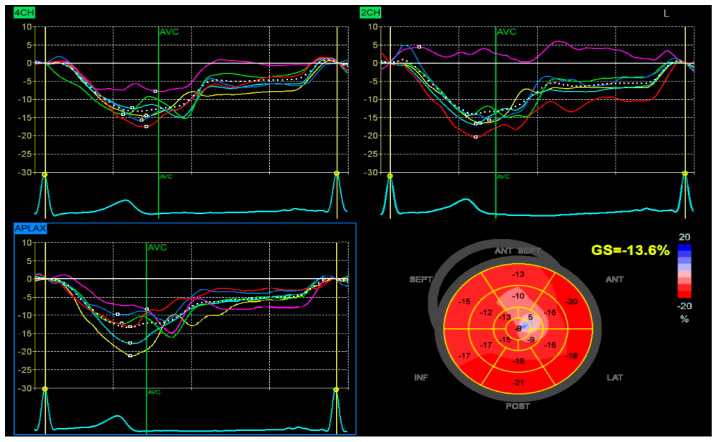
Speckle-tracking echocardiography demonstrating GLS analysis in apical four-chamber (4CH), two-chamber (2CH), and apical long-axis (APLAX) views. The bull’s-eye plot shows segmental strain reduction in the anterior and septal regions, with a calculated GLS of −13.6%.

Despite high-intensity lipid-lowering therapy, the patient did not reach the LDL-C target recommended (≤55 mg/dL). After one month, the patient returned for elective revascularization of the residual lesion in the marginal artery, which was successfully treated with the implantation of a drug-eluting stent. Also, a PCSK9 inhibitor was introduced, resulting in a marked and sustained reduction in LDL-C to 42 mg/dL. This lipid target was maintained in the long term, with favorable clinical evolution and no recurrent cardiovascular events. The patient also demonstrated significant recovery of left ventricular systolic function, with an improvement in ejection fraction from 38% during the acute phase to 50% at one-month follow-up, with a global longitudinal strain (GLS) of −13.6% (Figure 6).

This case underscores the importance of initiating intensive lipid-lowering therapy as early as possible in high-risk patients. Rapid and sustained control of LDL-C is critical not only for secondary prevention but also for improving long-term clinical outcomes. A timely, stepwise approach to lipid management—escalating therapy when needed—can ensure optimal cardiovascular protection, particularly in younger patients with premature or unstable atherosclerotic disease.

### 3.2. Case 2

**Figure 7:**
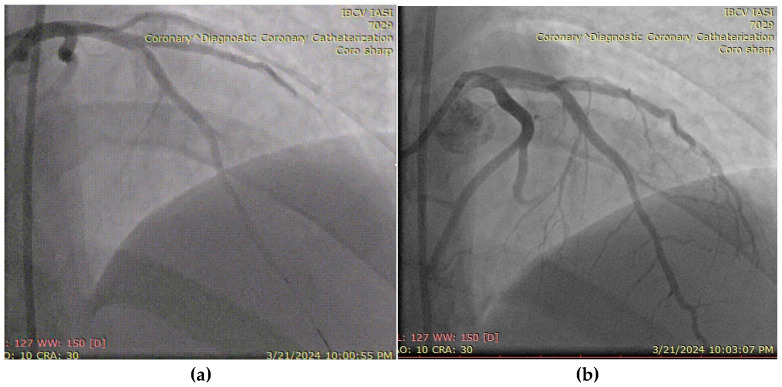
(**a**) Coronary angiography of the left coronary system (with subocclusive lesion at the left anterior descending artery). (**b**) Placement of a drug-eluting stent at that level.

A 27-year-old male, active smoker and recreational user of ecstasy and cocaine, presented with acute chest pain and was diagnosed with an antero-lateral ST-elevation myocardial infarction. He had no prior medical history or known comorbidities. Symptoms began following intense physical exertion and cocaine use. Upon admission, he was hemodynamically and respiratory stable, with a blood pressure of 120/75 mmHg, heart rate of 85 bpm, and oxygen saturation of 97%. Laboratory evaluation showed elevated cardiac enzymes and an LDL-C level of 102 mg/dL. Emergency coronary angiography revealed a subocclusive lesion in the left anterior descending artery, without other angiographic signs of atherosclerosis. The lesion was successfully treated with percutaneous coronary intervention and the placement of a drug-eluting stent (Figure 7). The patient was initiated on DAPT (aspirin and ticagrelor), high-dose statin (atorvastatin 80 mg/day), and a beta-blocker (bisoprolol 2.5 mg twice daily). LV ejection fraction was preserved at discharge, with a favorable early clinical outcome.

This case illustrates the unique presentation of AMI in a young adult with no traditional comorbidities but significant lifestyle-related risk factors. The absence of widespread atherosclerosis, the isolated critical lesion in a single coronary artery, and the triggering role of exertion and cold exposure in the context of recent drug use highlight the multifactorial and often nonatherosclerotic mechanisms of MI in this age group. Management of such cases is challenging due to the need for both acute revascularization and targeted secondary prevention strategies, including behavioral interventions and long-term cardiovascular risk modification.

### 3.3. Case 3

**Figure 8:**
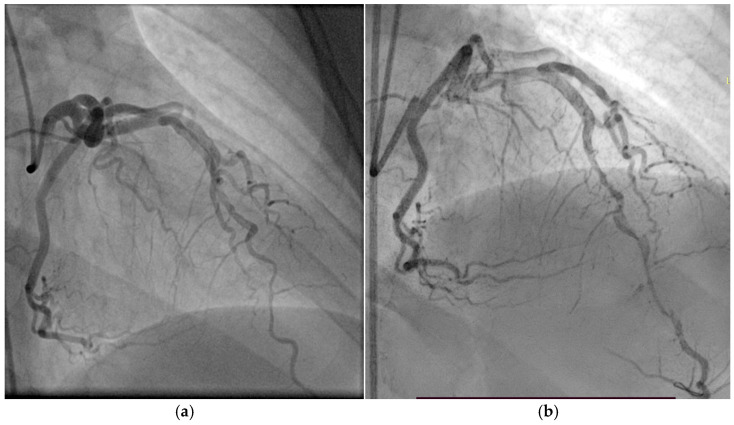
(**a**) Coronary angiography of the left coronary system (with SCAD at the left anterior descending artery). (**b**) Placement of the wire and after that the two drug-eluting stents at that level.

A 50-year-old hypertensive female was referred to our clinic following the onset of an inferior STEMI, with ventricular fibrillation at the debut which required four external electric shocks. On admission, she was hemodynamically and respiratory stable, with a blood pressure of 160/90 mmHg, heart rate of 85 bpm, and peripheral oxygen saturation of 97%. Laboratory analysis revealed elevated cardiac enzymes, an LDL-C level of 162 mg/dL, and a slight grade of normochromic, normocytic anemia (Hb = 10.8 g/dL), with other parameters within normal limits. Transthoracic echocardiography showed a preserved EF but with hypokinesis in the apical third of the postero-inferior wall, apex, and the apical half of the interventricular septum, with a global longitudinal strain (GLS) of −12.6%. Coronary angiography revealed one-vessel CAD, with SCAD in the third segment of LAD and a maximum stenosis of 70%, with the recommendation of conservative treatment (Figure 8a). During hospitalization, the patient again developed important chest pain, this time with the ECG aspect of an anterior STEMI, and emergency coronary angiography revealed the progression of SCAD with the complete occlusion of the vessel (Figure 8b), which required two active pharmacological stents placed at that level. At discharge, the patient maintained preserved EF, with a slightly circumferential pericardial effusion.

This case illustrates the dynamic and challenging nature of spontaneous coronary artery dissection management. Initially, a conservative approach was chosen, in accordance with current ESC and AHA/ACC guidelines, given the patient’s hemodynamic stability, preserved coronary flow, and limited dissection. This aligns with the preferred strategy for most SCAD cases, especially when there is no ongoing ischemia, as spontaneous healing is common and PCI carries a higher risk of complications. However, the patient’s subsequent anterior STEMI with angiographic progression to complete LAD occlusion necessitated urgent PCI with drug-eluting stents. While intravascular imaging techniques were considered, they were deferred in the acute phase due to concerns about further propagation of the dissection. The case underscores the importance of close monitoring, dynamic risk assessment, and flexibility in management strategy, balancing guideline recommendations with real-time clinical evolution.

### 3.4. Case 4

**Figure 9:**
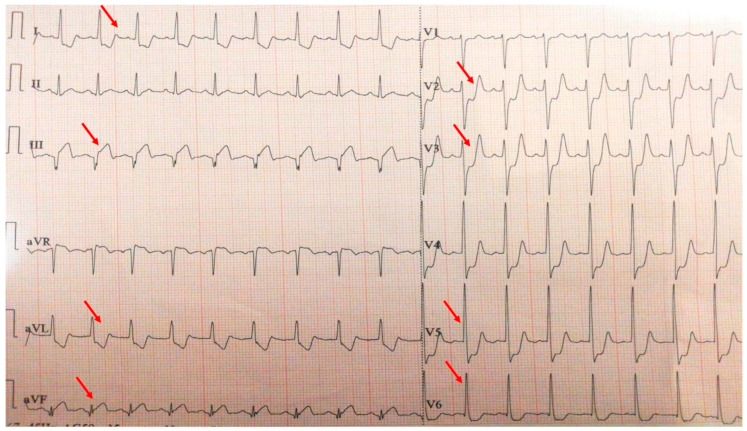
Electrocardiogram with postero-infero-lateral STEMI (red arrows).

A 35-year-old male with a known history of HIV infection diagnosed at the age of 20, under chronic antiretroviral therapy with lamivudine (a nucleoside reverse transcriptase inhibitor) and ritonavir (a protease inhibitor), was referred to our clinic following the onset of a postero-infero-lateral STEMI (Figure 9). The patient reported a six-month history of progressive exertional angina and dyspnea, which acutely worsened five hours prior to presentation. On admission, he was hemodynamically and respiratory stable, with a blood pressure of 100/60 mmHg, heart rate of 100 bpm, and peripheral oxygen saturation of 98%. Laboratory analysis revealed significantly elevated cardiac enzymes and an LDL-C level of 92 mg/dL, with other parameters within normal limits. Transthoracic echocardiography demonstrated a reduced left ventricular ejection fraction of 36% and a markedly impaired GLS of −10%.

**Figure 10:**
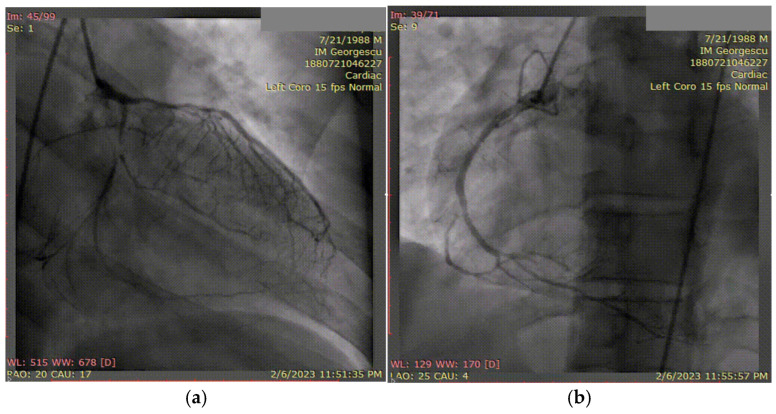
(**a**) Coronary angiography of the left coronary system. (**b**) Coronary angiography of the right coronary system. Coronary angiography showed severe three-vessel disease: 90% stenosis of the LAD and first diagonal branch, chronic proximal occlusion of the LCX, and 95% proximal stenosis of the RCA involving the posterior descending artery. A 30% distal stenosis of the LM artery was also noted.

Coronary angiography (Figure 10) revealed extensive three-vessel CAD, including a 90% stenosis of the LAD artery, 90% proximal stenosis of the first diagonal branch, chronic total occlusion of the proximal circumflex artery (LCX), and a 95% proximal occlusion of the right coronary artery (RCA) with involvement of the posterior descending artery. Additionally, a 30% distal stenosis of the LM coronary artery was noted. Due to the severity and complexity of the coronary lesions, CABG was recommended and successfully performed two months later.

This case underscores key considerations in the evaluation and management of myocardial infarction in a young adult with HIV infection. Notably, the patient developed extensive multivessel coronary artery disease in the absence of conventional cardiovascular risk factors, suggesting a significant role for HIV-related pathophysiological mechanisms. Chronic systemic inflammation, endothelial dysfunction, and the metabolic consequences of long-term antiretroviral therapy—particularly protease inhibitors—are known contributors to premature atherosclerosis in this population. The prolonged symptom duration prior to presentation raises the possibility of chronic myocardial ischemia, which was supported by the markedly reduced left ventricular ejection fraction and impaired global longitudinal strain on echocardiography. Given the diffuse nature and severity of coronary involvement, surgical revascularization was deemed the most appropriate therapeutic strategy. This case highlights the critical need for proactive cardiovascular risk assessment and individualized management in patients living with HIV, as well as the importance of a multidisciplinary approach in complex cases involving nontraditional risk profiles.

## 4. Implications for Clinical Practice and Future Directions

The growing incidence of AMI in young adults demands a paradigm shift in cardiovascular risk assessment and management strategies. These cases reveal the multifactorial nature of early-onset AMI, encompassing both traditional atherothrombotic processes and nonatherosclerotic mechanisms such as vasospasm, SCAD, coronary vasculitis, and substance-induced injury. Standard risk calculators often fail to capture the complexity and diverse risk profiles of younger individuals, underscoring the need for more tailored screening approaches that incorporate genetic predisposition, inflammatory states, and lifestyle-related exposures, including recreational drug use and HIV infection.

Clinical vigilance is essential, particularly in patients with atypical risk profiles or persistent symptoms despite young age. These cases highlight the need for early lipid profiling, expanded biomarker testing (including Lp(a) and apolipoproteins), and aggressive lipid-lowering strategies when indicated. Furthermore, tools such as intravascular imaging and strain echocardiography can assist in detecting vulnerable plaques and subclinical myocardial dysfunction, improving diagnostic precision and guiding therapy.

Future directions should focus on the development of age-specific guidelines for early recognition and management of AMI in young adults, including risk stratification algorithms that account for sex-specific and nontraditional risk factors. There is also an urgent need for large-scale, prospective studies exploring the long-term outcomes and optimal secondary prevention strategies in this unique patient population.

## 5. Conclusions

Acute myocardial infarction in young adults is an increasingly recognized clinical entity with a distinct pathophysiological spectrum compared to older populations. While premature atherosclerosis remains a common cause, a significant proportion of cases arise from nonatherothrombotic mechanisms, etiologies that necessitate a high index of suspicion and a tailored diagnostic and therapeutic approach.

Our findings underscore the importance of the early recognition of atypical presentations and nontraditional risk factors, especially in individuals without overt cardiovascular comorbidities. Advanced imaging modalities and comprehensive risk profiling—including lipid panels, thrombophilia screening, and autoimmune testing—play a critical role in establishing an accurate diagnosis.

Multidisciplinary collaboration, involving cardiology, internal medicine, rheumatology, infectious disease, and psychiatry specialists where appropriate, enhances care for this heterogeneous and vulnerable population. Future research should focus on identifying predictive biomarkers, understanding genetic predispositions, and evaluating long-term outcomes to refine prevention and treatment strategies in this emerging subgroup.

## Figures and Tables

**Table 1:** Diagnostic and treatment challenges of MI in young adults.

Etiology	Typical Patient	Mechanism	Key Diagnostic Test	Treatment
Premature atherosclerosis	FH, smoker	Plaque rupture or erosion	Lipid profile, genetic testing (FH), coronary angiography	Lifestyle changes, lipid-lowering medication, antiplatelet therapy
Coronary vasospasm	Young males, often with substance use (e.g., cocaine) or smoking history	Vasospasm, thrombosis	Toxicology, coronary angiography ± provocative testing (ergonovine, acetylcholine)	Smoking/substance cessation, calcium channel blockers, nitrates
Spontaneous coronary artery dissection	Young women, often peripartum or under emotional/physical stress	Intimal tear or intramural hematoma	Coronary angiography with IVUS or OCT for confirmation	Conservative management preferred; PCI if ongoing ischemia or instability
Coronary artery vasculitis	Patients with systemic autoimmune or inflammatory disease	Immune-mediated inflammation	Inflammatory markers, autoantibodies, coronary angiography	Treatment of underlying condition, immunosuppressive therapy, lipid-lowering medication

FH, familial hypercholesterolemia; IVUS, intravascular ultrasound; OCT, optical coherence tomography; PCI, percutaneous coronary intervention.

## Data Availability

All data generated or analyzed during this study are included in the article.

## Abbreviations

The following abbreviations are used in this manuscript:

2CH	Apical 2-chamber view
4CH	Apical 4-chamber view
ACEIs	Angiotensin-converting enzyme inhibitors
ACS	Acute coronary syndrome
AMI	Acute myocardial infarction
APLAX	Apical long-axis view
ARBs	Angiotensin receptor blockers
ART	Antiretroviral therapy
ASCVD	Atherosclerotic cardiovascular disease
CABG	Coronary artery bypass grafting
CAD	Coronary artery disease
CAV	Coronary artery vasculitis
CCTA	Coronary computed tomography angiography
CVD	Cardiovascular disease
DAPT	Dual antiplatelet therapy
FMD	Fibromuscular dysplasia
GLS	Global longitudinal strain
HDL-C	High-density lipoprotein cholesterol
HF	Heart failure
HIV	Human immunodeficiency virus
IVUS	Intravascular ultrasound
LAD	Left anterior descending coronary artery
LCX	Left circumflex coronary artery
LDL-C	Low-density lipoprotein cholesterol
LM	Left main coronary artery
Lp(a)	Lipoprotein(a)
LV	Left ventricle
LVEF	Left ventricular ejection fraction
MACCE	Major adverse cardiac and cerebrovascular events
MCP-1	Monocyte chemoattractant protein-1
MI	Myocardial infarction
MINOCA	Myocardial infarction with nonobstructive coronary arteries
NNRTIs	Non-nucleoside reverse transcriptase inhibitors
NRTIs	Nucleoside reverse transcriptase inhibitors
NSTEMI	Non-ST-elevation myocardial infarction
OCT	Optical coherence tomography
P-SCAD	Pregnancy-associated spontaneous coronary artery dissection
PCI	Percutaneous coronary intervention
PLWH	People living with HIV
RCA	Right coronary artery
SCAD	Spontaneous coronary artery dissection
SpO2	Oxygen saturation
STEMI	ST-elevation myocardial infarction
T1M1	Type 1 myocardial infarction
T2M2	Type 2 myocardial infarction
TCFA	Thin-cap fibroatheromas
